# Fouling of an anion exchange chromatography operation in a monoclonal antibody process: Visualization and kinetic studies

**DOI:** 10.1002/bit.24898

**Published:** 2013-03-31

**Authors:** Edward J Close, Jeffrey R Salm, Timothy Iskra, Eva Sørensen, Daniel G Bracewell

**Affiliations:** 1 The Advanced Centre for Biochemical Engineering, Department of Biochemical Engineering, University College LondonTorrington Place, London, WC1E 7JE, UK; 2 Centre for Process Systems Engineering, Department of Chemical Engineering, University College LondonTorrington Place, London, UK; 3 PfizerAndover, Massachusetts

**Keywords:** chromatography resin fouling, scanning electron microscopy, confocal laser scanning microscopy, anion exchange chromatography, therapeutic protein

## Abstract

Fouling of chromatographic resins over their operational lifetimes can be a significant problem for commercial bioseparations. In this article, scanning electron microscopy (SEM), batch uptake experiments, confocal laser scanning microscopy (CLSM) and small-scale column studies were applied to characterize a case study where fouling had been observed during process development. The fouling was found to occur on an anion exchange (AEX) polishing step following a protein A affinity capture step in a process for the purification of a monoclonal antibody. Fouled resin samples analyzed by SEM and batch uptake experiments indicated that after successive batch cycles, significant blockage of the pores at the resin surface occurred, thereby decreasing the protein uptake rate. Further studies were performed using CLSM to allow temporal and spatial measurements of protein adsorption within the resin, for clean, partially fouled and extensively fouled resin samples. These samples were packed within a miniaturized flowcell and challenged with fluorescently labeled albumin that enabled in situ measurements. The results indicated that the foulant has a significant impact on the kinetics of adsorption, severely decreasing the protein uptake rate, but only results in a minimal decrease in saturation capacity. The impact of the foulant on the kinetics of adsorption was further investigated by loading BSA onto fouled resin over an extended range of flow rates. By decreasing the flow rate during BSA loading, the capacity of the resin was recovered. These data support the hypothesis that the foulant is located on the particle surface, only penetrating the particle to a limited degree. The increased understanding into the nature of the fouling can help in the continued process development of this industrial example.

Scanning electron microscopy (SEM), batch uptake experiments, confocal laser scanning microscopy (CLSM) and small-scale column experiments were applied to characterize a case study where fouling had been observed on an anion exchange chromatography in a monoclonal antibody process. The results suggest the foulant is located on the particle surface, resulting in a minimal decrease in saturation capacity, but having a significant impact on the kinetics of adsorption, severely decreasing protein uptake rate.

## Introduction

Fouling of chromatographic resin over operational lifetimes can be a serious problem associated with industrial separations. This phenomenon, attributed to repeated or prolonged exposure to the complex mix of components commonly seen in feed streams, can significantly reduce binding capacities and alter process kinetics (Staby et al., 29). The fouling may have an impact on the ability of the process to deliver product that meets pre-determined acceptance criteria, or reduce the lifetime of the resins which in turn has an impact on the cost of manufacture. Although standard clean-in-place (CIP) procedures can help restore columns towards their original state, fouling by certain types of material is often irreversible under typical conditions (Jin et al., 16), and this must be balanced by the fact that more stringent cleaning can cause degeneration of the resin pore structure and ligand (Jiang et al., 14; Muller-Spath et al., 23). Fouling therefore limits the number of available cycles before a column must be replaced, thus increasing the expense of downstream processing, which can account for up to 60% of total process cost of goods (Kelley, 17). Furthermore, there is an increasing desire to develop more fundamental understanding of processes under the Quality by Design paradigm (FDA, 8,9).

Against this background, the characterization of fouling is an extremely important issue, but in spite of this, relatively few systematic investigations of packed bed chromatography column fouling have been reported in the literature. The conventional approach examines a range of performance indicators such as pressure drop profiles, dynamic capacity, and breakthrough curves where scaled down columns are repeatedly loaded with fouling material (Boushaba et al., 3; Bracewell et al., 4; Chau et al., 5; Shepard et al., 26). Rather than considering fouling directly, studies often focus on related subjects such as resin lifetime issues and CIP procedures (Muller-Spath et al., 23; Norling et al., 24). Recently, confocal laser scanning microscopy (CLSM), a tool that can monitor adsorption processes on a particle scale by observing the distribution of a fluorescent molecule within particles (Ljunglöf and Hjorth, 21; Ljunglöf and Thömmes, 22; Linden et al., 20; Hubbuch et al., 12), has been utilized to visualize fouling at the particle level (Jin et al., 16; Siu et al., 27–28). This has provided valuable insight into the relationship between foulant location and the resulting processing effect.

In this work, we consider a case study where resin fouling had been observed during process development; that of an industrial anion exchange (AEX) polishing step following a protein A affinity capture step in a process for the purification of a monoclonal antibody (mAb). The anion-exchange chromatography, which operates in weak-partitioning mode (Kelley et al., 18), was characterized through high throughput screening experiments (Coffman et al., 6; Kelley et al., 19), as well as in-house cycling studies performed on qualified scale-down models, and large-scale manufacturing runs. The AEX resin has been successfully used as part of a two column platform process for the purification of numerous monoclonal antibodies in the past (Kelley et al., 18), with no significant fouling phenomena observed. Hence it was surprising that for this protein as column lifetime increased, when protein A elution pool material was loaded onto the AEX resin, significantly earlier breakthrough of impurities and premature loss of capacity was observed. Interestingly, it was found that the lifetime of the AEX resin was linked to the protein A cycle number such that as protein A cycle number increased, there was a consequent increase in capacity of the AEX polishing step. The data suggested a unique quality of the particular feed stream resulted in the fouling. Iskra et al. also found that the fouling could be accelerated by overloading the AEX resin well beyond normal operating conditions. Different control strategies were considered for preventing impurity breakthrough and improving resin lifetimes. An investigation using small-scale chromatography, dynamic light scattering, mass spectroscopy and Fourier transform infrared spectroscopy (FTIR), indicated that the most likely hypothesis was that resin was being fouled by a combination of product and host cell proteins. A detailed account has been presented in the literature (Iskra et al., 13). In this article, we aim to elucidate on this resin fouling case study, by revealing the location of the foulant, and determining the mechanistic effects fouling has on protein uptake kinetics and resin capacity.

We apply scanning electron microscopy (SEM), batch uptake experiments, CLSM on a miniaturized packed bed, and small-scale column experiments to samples of fouled resin derived from the industrial process using the worst case feed stream and overloading conditions. SEM and batch uptake experiments are used to give initial indications of foulant location and resin performance as fouling progresses, before CLSM is used to conduct a more detailed investigation. TexasRed labeled BSA is used as a reporter molecule for protein uptake kinetics. The technique uses a flowcell to measure changes at various stages of fouling in resin capacity and uptake kinetics, at a particle level. The time and space distribution of the labeled BSA within the resin particles is recorded in situ in order to facilitate a comparison between clean, partially fouled and extensively fouled resin. Finally, column studies are conducted to investigate the effect of the foulant on protein uptake and breakthrough performance of a column system. Together these techniques (summarized in Table I) enable us to determine the spatial location of the foulant and its effect on the process during protein uptake.

**Table I:** Experimental methodology for investigating clean, partially found, and extensively fouled resin samples

Experiment	Results	Purpose
Batch uptake	Uptake curves	Initial indication of impact of fouling on uptake rate and saturation capacity
Scanning electron microscopy	Images of particle surfaces	Morphology of resin surface
Confocal laser scanning microscopy (CLSM) during live uptake	Radial light intensity profiles of a BSA reporter molecule during uptake in a miniature column	Fouling effect on intra-particle profiles of bound BSA reporter molecule during uptake
Column studies	BSA reporter molecule breakthrough curves and dynamic binding capacities	Fouling effect on BSA reporter molecule breakthrough and dynamic binding capacity

## Materials and Methods

### Chemicals

All chemicals were purchased from Sigma-Aldrich (Dorset, UK) and were of analytical grade unless stated otherwise.

### Chromatography Resin and Equipment

MabSelect Protein A affinity chromatography resin was obtained from GE Healthcare (Uppsala, Sweden). Fractogel® EMD TMAE HiCap (M) AEX resin was obtained from EMD Merck (Darmstadt, Germany). All laboratory experiments were carried out using an ÄKTA FPLC chromatography system from GE Healthcare.

### Proteins

The mAb used in these studies was humanized IgG1 produced in recombinant Chinese hamster ovary (CHO) cells grown in serum free medium. Downstream processing prior to the AEX step considered in this work consisted of centrifugation and depth filtration, followed by protein A chromatography. Bovine serum albumin (BSA)—Texas Red(R) conjugate was purchased from Invitrogen (Paisley, UK).

### Protein A Chromatography

The column used in protein A chromatography was 1.6 cm in diameter and 30 cm in height. The column was equilibrated with 0.15 M sodium chloride at pH 7.5 prior to loading. Clarified condition media was then applied followed by a two column volumes (CV) wash of the equilibration buffer. This was followed by 5 CV's of 1.8 M calcium chloride at pH 7.5. The elution pool consisted of material collected from start in UV rise, to a total of 2.5 CV's, collected as the process pool. The remaining bound protein was removed using an additional 5 CV's of low pH followed by sanitization with 50 mM NaOH, 0.5 M sodium sulfate, and stored in 16% ethanol, 150 mM NaCl, 50 mM TRIS, pH 7.5.

### Anion Exchange Chromatography

The AEX columns used in this study were 0.5 cm in diameter and either 5 or 15 cm in height, and were operated in weak partitioning mode (Iskra et al., 13; Kelley et al., 18). The columns were equilibrated with 50 mM TRIS, 10 mM NaCl at pH 8.1. Protein A peak pools were applied to the column at 150 cm/h followed by a 3 CV wash of the equilibration buffer. Protein A peak pools contained the product of interest, host cell protein, DNA and residual protein A which had leached from the affinity capture resin, and approximately 3.5% high molecular mass species (HMMS). The turbidity of the protein A pool was 28.1 NTU (Iskra et al., 13). The load eluate and wash volumes were collected together as the process pool, and any remaining bound protein was removed using a 2 M NaCl strip buffer. The columns were sanitized with 2 M NaCl, 0.5 M NaOH and stored in 16% ethanol, 150 mM NaCl, 50 mM TRIS, pH 7.5. The loading conditions used during column runs (50 mM TRIS, 10 mM NaCl at pH 8.1) had been determined by high-throughput screening (HTS) under batch binding conditions, and were confirmed using scale—down column chromatography experiments to provide sufficient clearance of impurities (residual HMMS <1.5%, HCP clearance >3.0 LRV, and leached protein A clearance >3.0 LRV), while maintaining yield >90%, prior to resin fouling. These conditions would be expected to produce the desired product quality in large-scale manufacturing (Iskra et al., 13).

### Generation of Fouled Resin Samples

Three resin samples were used in subsequent experimental studies to characterize the fouling. These were generated by conducting multiple cycles of the AEX chromatography on a column 0.5 cm in diameter and 15 cm in height, using the worst case feed stream and overloading conditions (Iskra et al., 13), according to the standard operating procedure set out previously. The three resin samples are classified as follows: unused clean resin, fully fouled resin representative of resin at the end of the column's lifetime (hereon referred to as extensively fouled resin), and partially fouled resin, representative of an intermediate state of fouling.

### Batch Uptake Experiments

A set amount of TMAE HiCAP (M) resin was allowed to settle by gravity. After measuring the settled volume, the resin was washed with ultra-pure Millipore water to remove the storage ethanol solution and then equilibrated with 0.05 M TRIS Base pH 9.0 HCl adjusted buffer, giving a final concentration of 50% (v/v). Fifty microliters of this slurry was then aliquoted to a 2 mL eppendorf tube. Adsorption was started by adding 2 mL of Texas Red labeled BSA to the resin sample (Overall BSA concentration: 5 mg/mL, dye to protein (D/P) ratio: 0.01). The eppendorf was kept under constant agitation, except at fixed times when the eppendorf tube was quickly centrifuged for 10 s at 1,200*g*, before a 50 µL sample was taken from the supernatant and collected for subsequent UV analysis at 280 and 593 nm by a Nanodrop. The sedimented resin particles were quickly resuspended by resuming agitation. For the duration of the experiment resuspension was ensured by placing the eppendorf tube onto an orbital shaker rotating at 2,000 rpm, and confirmed by visual inspection.

### Scanning Electron Microscopy (SEM)

Sample preparation for SEM consisted of sample drying followed by gold coating. A thin layer of resin slurry was pipetted onto a glass slide which had been pre-coated in gold and mounted onto a copper block. Excess liquid was carefully adsorbed on filter paper without contacting the resin particles, before the sample was left for 30 min to allow any remaining ethanol to evaporate. The dried sample was thereafter transferred to a high resolution ion beam coater (Gatan Model 681, Oxford, UK), and ion sputtered with gold at an angle of 45° in order to form a 2–3 nm gold layer on the surface of the resin particles. The ion beam coater was operated at 6 mA at an acceleration voltage of 10 keV. Coated surfaces were subsequently imaged with a JEOL JSM-7401F scanning electron microscope (JEOL Ltd, Tokyo, Japan) at 1 keV accelerating voltage.

### Live Uptake Imaging by Confocal Laser Scanning Microscopy (CLSM)

Image acquisition was performed on an inverted confocal laser scanning microscope (Leica TCS SPEinv, Leica Microsystems GmbH, Mannheim, Germany) equipped with krypton/argon (*λ* = 488 nm and *λ* = 568 nm) and helium/neon (*λ* = 633 nm) lasers. Using a 40× oil immersion objective, images (512 × 512) were captured (3 averages) through the Leica Application Suite (LAS) software (Version 2.0) (Leica Microsystems GmbH). Optimal laser settings, including laser intensity, signal gain, offset and emission detection range were determined to ensure that there were no auto fluorescence effects, and that even at full particle saturation the detected emitted light intensity stayed within the confocal laser scanning microscope's detection range. The settings were then kept constant for the duration of the study.

In order for BSA to be detected by the confocal laser scanning microscope, it must be labeled by a suitable fluorescent dye molecule (Ljunglöf and Thömmes, 22). There are many different dye molecules currently available for this purpose. However, it has been reported that the attachment of dye molecules can significantly change the adsorption behavior of the BSA and therefore must be carefully selected (Hubbuch and Kula, 11; Teske et al., 32). BSA conjugated to TexasRed, AlexaFluoro488, Cy3, and Cy5 on a strong anion exchanger chromatographic system, similar to that considered in this work, has previously been screened to determine which was most suitable for CLSM (Susanto et al., 31). The elution profiles of the conjugates were compared with native BSA in relation to retention time and peak shape. BSA TexasRed conjugate showed the least deviation from the native BSA, and was therefore selected in this work. The ratio between native and TexasRed conjugated BSA was tested by measuring UV adsorption at 280 and 593 nm throughout the batch uptake experiments. The constant ratio during protein uptake over 1 h confirmed that there were minimal competitive effects in our system.

In order to minimize the readsorption of emitted fluorescence by other dye molecules, a dye to protein ratio (D/P) of 0.01 was used for the feed solution following recommended literature D/P ratios (Hubbuch and Kula, 11). At this D/P ratio we were able to assume that the contribution of emitted fluorescence readsorption to light attenuation could be neglected.

Control experiments investigating fluorophore bleaching upon repeated use were completed, and confirmed that bleaching could be neglected for the purposes of this work. A range of precautions were taken to minimize fluorophore bleaching throughout the experimental work: the use of low laser powers, minimized exposure times and wrapping all samples in aluminum foil in order to minimize exposure to light during transition and storage.

**Figure 1:**
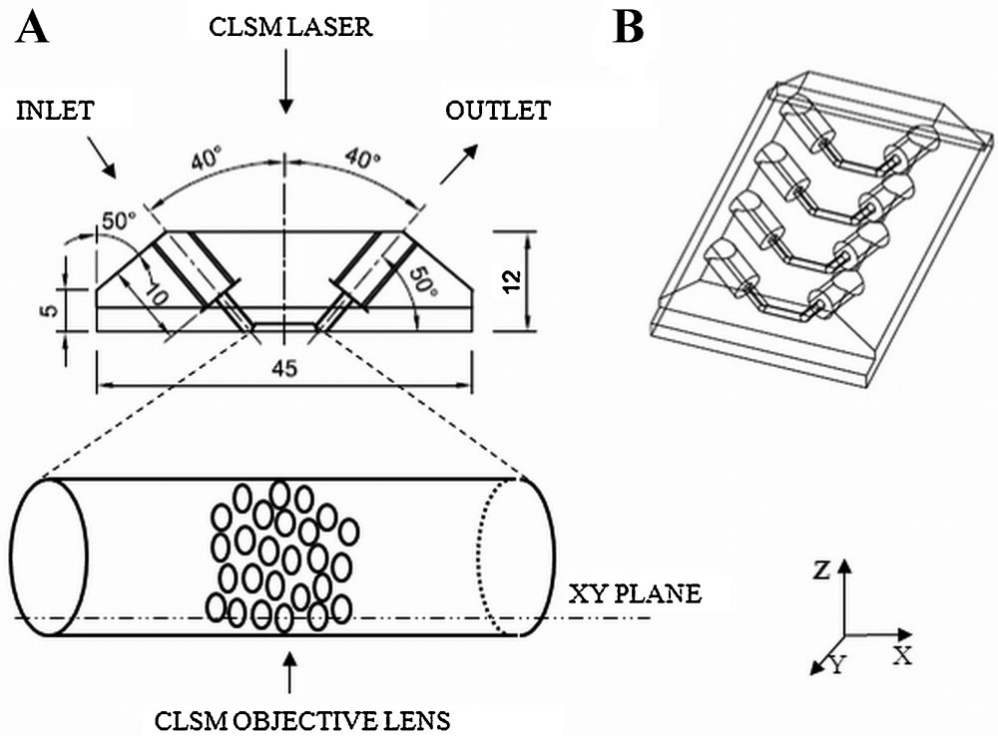
A: Side elevation of the miniaturized flowcells setting within the confocal laser scanning microscope. XY image acquisition plane shown on diagram. B: Three-dimensional representation of the miniaturized flowcell used for live imaging of the intra-particle uptake within a packed bed.

A miniaturized flowcell was fabricated similar in design to that used by Hubbuch and Kula (11). Four horizontal channels (10 mm length, 1 mm diameter) were drilled into a Pyrex block with 45° inlets on both sides. A viewing window was then created by fixing a cover slip onto the open face of the block with epoxy glue Araldite® (Huntsman Advanced Materials, Cambridge, UK) in order to seal each channel. The resulting effective column volume was 0.02 mL. A schematic of the flowcell is shown in Figure 1.

Flowcell channels were packed by manually administering resin slurry (50%, v/v) from a syringe. Great care was taken to ensure that the resin was not over or under packed. Frits were placed at either end of the channel, which was then connected to a syringe pump. The resin was washed with ultra-pure Millipore water to remove the storage ethanol solution (2 mL, 150 cm/h), before equilibration with 0.05 M TRIS Base pH 9.0 HCl adjusted equilibration buffer (2 mL, 150 cm/h).

Texas Red labeled BSA feed was adjusted to the required pH and salt concentration in the running buffer, and loaded onto the resin bed within the flowcell channel at 150 cm/h for 90 min. Images were recorded using a Confocal Laser Scanning Microscope at set time intervals with excitation at 568 nm and emission detection in the range 550–701 nm. The setting of the flowcell within the laser scanning confocal microscope is shown in Figure 1.

### Live Uptake Data Processing

**Figure 2:**
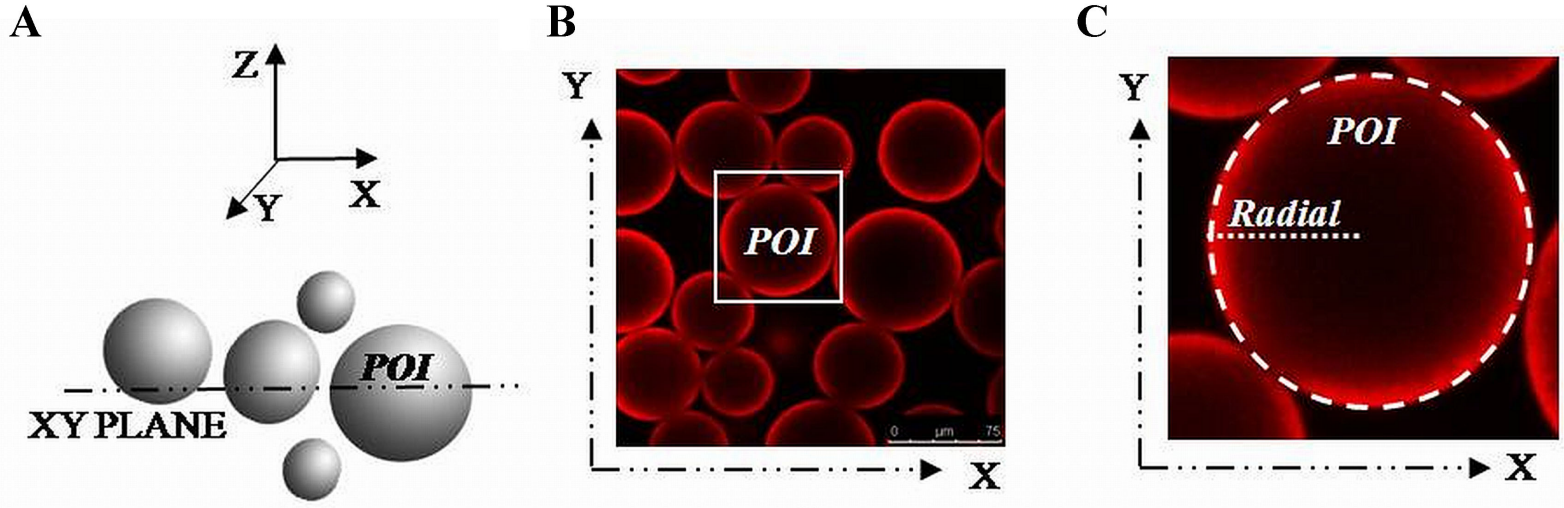
A: The XY plane intersecting with center of the particle of interest (POI) is selected. B: The POI's center XY plane image from CLSM, also showing all available particles in the flowcell area imaged. C: Illustrating how the emitted fluorescence intensity is measured as a function of radial coordinates (dotted line), and is averaged over particle circumference (dashed line).

The large number of confocal images from the flowcell experiments were processed to generate a reliable set of radial light intensity profiles. This was done in order to allow a direct comparison of the spatial location of BSA within resin particles during protein uptake between the clean, partially fouled and extensively fouled samples over the 90-min experiments. For each experimental data set five particles were selected for data processing from the area of the flowcell imaged. We found that using more than five particles gave negligible benefits in terms of the reliability of our data. The appropriate XY image where the focal plane intersected with the center of each particle was then selected at each time interval, as illustrated in Figure 2A. This was possible because at each time interval throughout the 90-min flowcell experiments, images of XY planes of the flowcell bed area under scrutiny were taken over a range of *z* values at 5 µm intervals. The selection of five particles from those available (up to 14) also helped us to ensure this center cross-section positioning, as we were able to only select particles where the focal plane exactly intersected with the center of each particle.

The next step was to generate radial profiles of the emitted light intensity. This is typically done by a simple linear profile evaluation through the central cross section of a scanned particle (Hubbuch and Kula, 11). However, this method neglects the inhomogeneous nature of protein uptake due to effects such as particle contact points and fouling. In this work, the emitted fluorescence intensity was measured as a function of the radial coordinate and subsequently averaged over particle circumference in order to account for this inhomogeneous uptake (illustrated in Fig. 2C). We utilized ImageJ v1.31 for this purpose, which is an imaging software developed by the Research Services Branch of the National Institute of Mental Health in Bethesda, MD, USA and is freely available in the public domain (Abramoff et al., 1; Rasband, 25).

The radial profiles of each particle were then normalized by dividing the radial dimension by the appropriate particle diameter. In this work the particle–fluid phase boundary was identified by the highest emitted light intensity across the radial profile, and subsequently used to calculate the particle diameter. It was found that the emitted light intensity values outside the determined particle–fluid phase boundary consistently dropped to an insignificant value within 4 µm of the particle diameter over the range of particle diameters analyzed (50–90 µm), for all resin samples. This is in agreement with the literature, where lengths of this region typically fall between 2 and 10 µm (Dziennik et al., 7; Susanto et al., 30). All emitted light intensity data were corrected for light attenuation effects following the methodology set out by Susanto et al. (30). Lastly, the corrected emitted light intensity radial profiles (normalized by particle diameter and averaged over particle circumference) were averaged over the five particles per resin sample. The resulting set of data thus describes the average time and space distribution of the BSA in the resin samples throughout the 90-min flowcell experiment.

### Column Studies

An iterative procedure where a column 0.5 cm in diameter and 5 cm in height was subjected to multiple AEX chromatography cycles using representative load material and a scheme of over challenge was performed, until the cumulative amount of protein from protein A peak pools that had been challenged onto the resin reached predefined amounts (1, 2, 3, 4, and 5 g/mL). Each time the cumulative load challenge reached one of the predefined amounts, the cycle in progress was allowed to run to completion, that is, the column washed, eluted, sanitized, and placed into storage buffer according to the methodology set out previously (Materials and Methods: AEX chromatography), and the AEX cycling paused. The effect of resin fouling that had occurred during the AEX cycling was then measured via full breakthrough of BSA at different flowrates (0.49, 0.33, 0.16, 0.08 mL/min). BSA breakthrough was conducted as follows: The column was equilibrated with 50 mM TRIS, 10 mM NaCl at pH 9.0. BSA was then applied to the column at the specified flowrate (load concentration 10 mg/mL, load challenge 300 mg/mL). Any bound BSA was then removed using a 50 mM TRIS, 2 M NaCl, pH 7.5 elution buffer. The column was further cleaned with 2 M NaCl, 0.5 M NaOH, and then placed in storage buffer (16% ethanol, 150 mM NaCl, 50 mM TRIS, pH 7.5). Once the breakthrough of BSA had been recorded at each flowrate, the column was returned to AEX chromatography cycling.

## Results and Discussion

The overall objective of this work was to determine the location of fouling on resin particles and the effect of this fouling on protein kinetics and resin capacity in an AEX polishing step from an industrial purification process. In the following, we first present the results from SEM imaging and batch uptake experiments which give initial indication of the foulant location and the progressive nature of the effect with cycle number. Following this, we present results from a detailed CLSM investigation. This includes the intra-particle radial adsorption profiles during protein uptake within the miniaturized flowcell bed, accompanied by corresponding uptake curves. Finally, we present the results from column studies which include BSA breakthrough profiles and dynamic binding capacities over a range of flowrates with increasing cycle number and hence also increasing fouling.

### Batch Experiments

**Figure 3:**
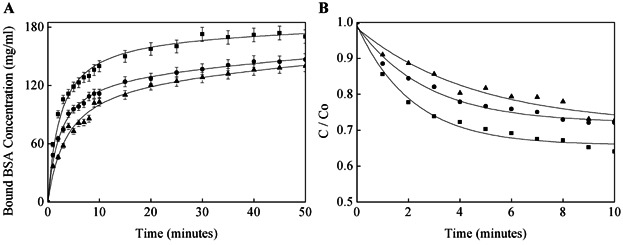
Batch uptake curves of 5 mg/mL BSA at 0.05 M TRIS Base pH 9.0, by clean ▪, partially fouled •, and extensively fouled ▴ Fractogel® EMD TMAE HiCap (M) resin particles during batch experiments. (Feed to resin volume ratio 80:1 (80×).) A: Bound BSA concentration as a function of time. B: Normalized bulk mobile phase BSA concentration as a function of time.

The batch experiments were designed to give an initial indication of the effect that the foulant had on protein uptake, and to confirm that there were no competitive effects in the system due to modification of BSA binding characteristics when conjugated with the TexasRed flurophore, in preparation for the CLSM study. Figure 3 shows the batch uptake curves of BSA with clean, partially fouled and extensively fouled resin samples. For all samples the batch adsorption kinetics were consistant with previous literature results on tentacle exchangers (Almodóvar et al., 2; Urmann et al., 33). However, a clear difference in the initial uptake rates was observed with clean resin having the fastest uptake, followed by partially fouled resin, then extensively fouled resin. More detailed analysis of the data indicated that the initial uptake of BSA by clean resin was roughly twice as fast as uptake by extensively fouled resin, indicating fouling significantly impacts mass transfer.

Figure 3 shows that by the end of the experiment equilibrium had not been reached by any of the resin samples. Although the amount of BSA bound to the extensively fouled resin was lower than the amount bound to the clean resin (partially fouled resin ∼87% amount bound to clean resin, extensively fouled resin ∼82% amount bound to clean resin), uptake was still ongoing. Firm conclusions regarding the effect that the foulant had on the saturation capacity of the resin could therefore not be made. As there was a constant ratio between conjugated and non-conjugated BSA in the supernatant throughout uptake, confirmation that there were no competitive adsorption effects in the system was achieved (not shown).

### Scanning Electron Microscope Imaging

**Figure 4:**
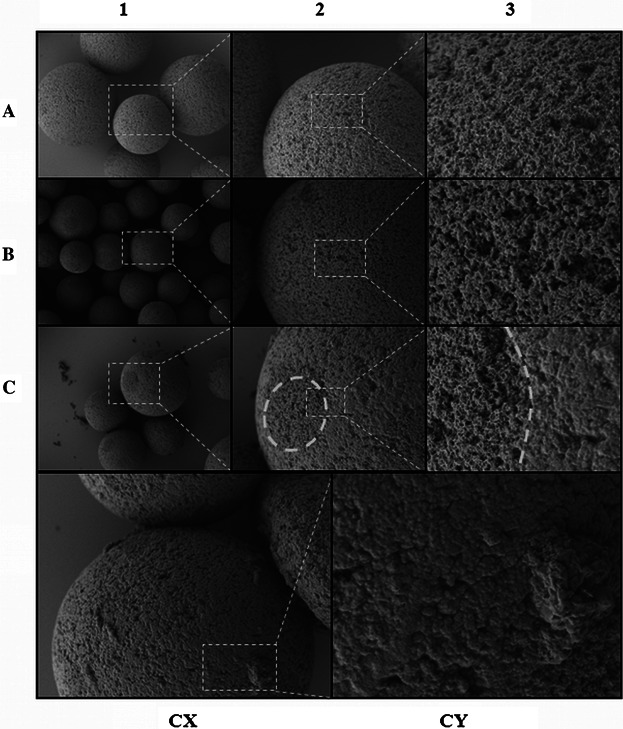
Scanning electron microscopy images of A: clean, B: partially fouled. C: Extensively fouled Fractogel® EMD TMAE HiCap (M) resin particles. A1: 1,000×, B1: 450×, C1: 750×, A2/C2: 3,000×, B2: 2,000×, A3/B3/C3: 10,000×, CY: 2,000×, CY: 7,000×.

Figure 4 shows high resolution SEM images of clean, partially fouled and extensively fouled resin particles at progressively higher magnifications. The area of the particle under analysis was kept constant for each sample. The images show distinct differences in the particle surface morphology between the three samples. In the clean resin (Fig. 4A), the surface is well-defined, homogenous, and the pores are clearly accessible. By comparison, the extensively fouled resin (Fig. 4C) shows a high amount of pore blockage by a material covering much of the surface. This is particularly clear on the highest magnification image (10,000× for A3/B3/C3). In Figure 4, images CX and CY illustrate the magnitude of the fouling, with many particles showing completely clogged pore entrances over a significant percentage of particle surface area. Fouling on partially fouled resin surface is not as obvious, but does show an intermediate level of pore blockage (Fig. 4B).

Interestingly, circular patches were found on the extensively fouled resin which were comparable in surface morphology to that seen in the clean resin images in Figure 4C. These are regions where particle–particle contact occurs within the packed bed, and show little or no fouling. Previous studies have also shown such areas, clearly distinguishable from the rest of the particle surface, and have reported localized external mass transfer resistance through these regions (Hubbuch et al., 12; Jin, 15; Siu et al., 28). In addition to the pore blocking, images CX and CY show larger pieces of clumped material on the particle surface that were common throughout fouled and partially fouled particles. The SEM imaging suggests a mechanism where the foulant blocks pore entrances but does not penetrate a significant distance into the particle, instead continuously growing outwards over successive cycles. This is in agreement with results by Jin (15) who reported the progressive build up of lipid based foulant on the surface of Sepharose® Butyl-S 6 Fast Flow resin over successive cycles.

### Live Uptake Experiments

**Figure 5:**
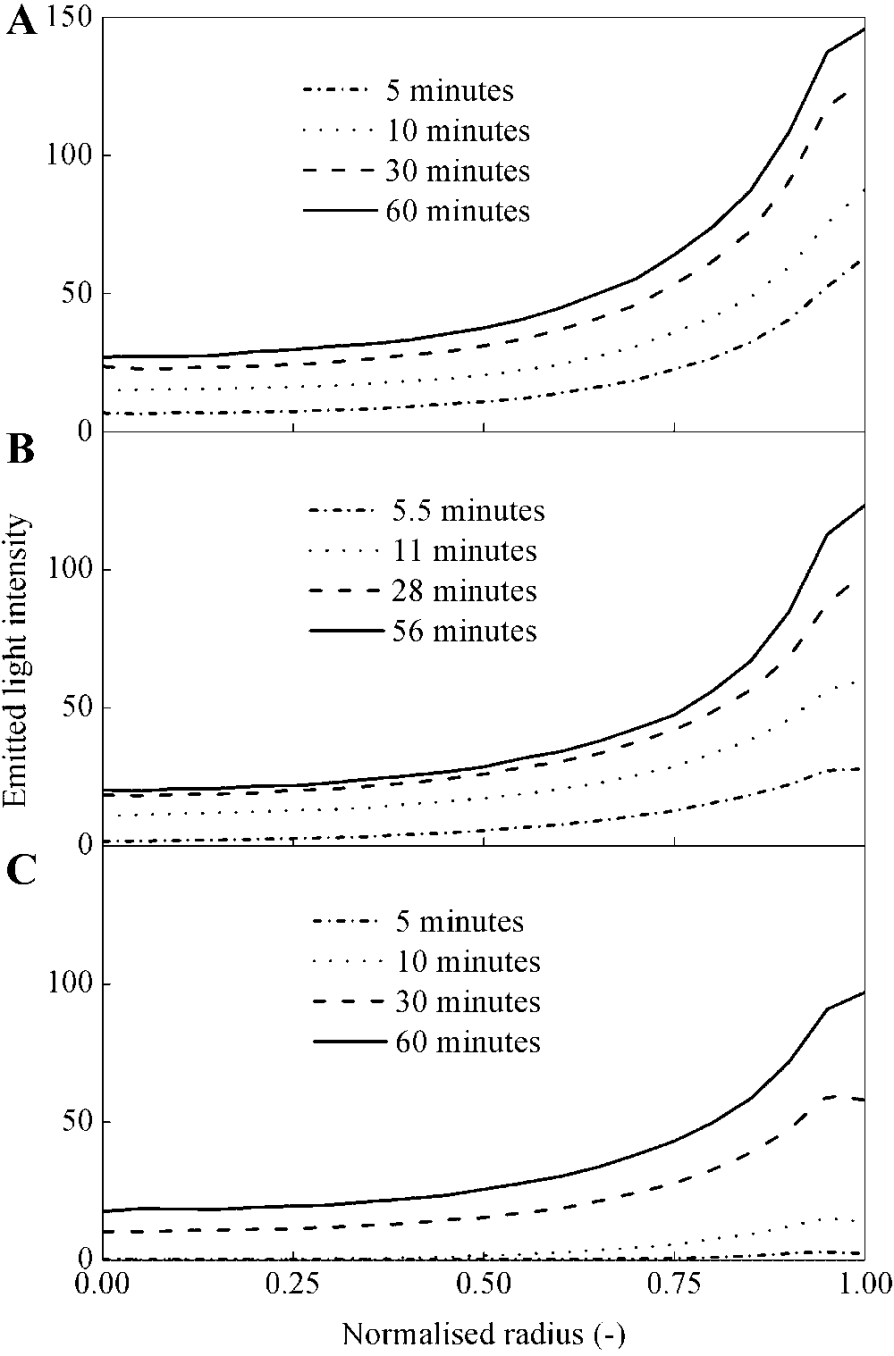
Average radial emitted light intensity of BSA (stationary phase: (A) clean, (B) partially fouled, (C) extensively fouled Fractogel® EMD TMAE HiCap (M) resin particles) over time (0, 5, 10, 30, 60 min), during the uptake of Texas Red labeled BSA from the process feed (5 mg/mL BSA, D/P ratio = 0.01, 0.05 M TRIS Base pH 9.0 HCl adjusted, 150 cm/h), during flowcell experiments.

**Figure 6:**
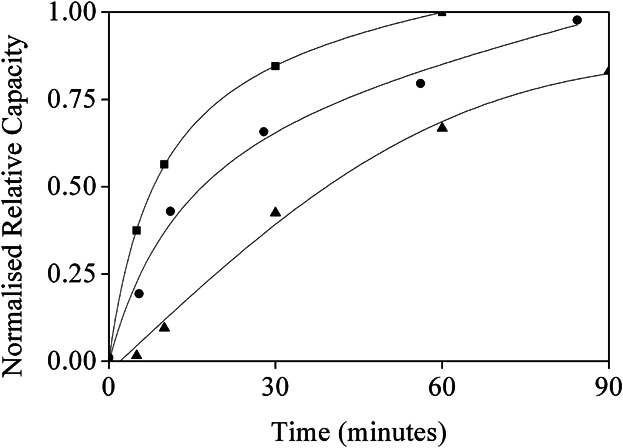
Relative uptake curves of clean ▪, partially fouled •, and extensively fouled ▴ Fractogel® EMD TMAE HiCap (M) resin calculated from integrating under intra-particle labeled BSA profiles during uptake in a packed bed in Figure 5, and correcting for the spherical nature of resin particles.

The purpose of the live uptake experiments was to conduct a direct comparison between the clean, partially fouled, and extensively fouled resin based on their intra-particle radial light intensity profiles, during uptake of a BSA reporter molecule in a packed bed using CLSM. The light intensity is proportional to the concentration of bound protein, and therefore can be used to estimate differences in the intra-particular mass transfer and adsorption (Fig. 5). Integrating the area underneath the radial light intensity profiles, and correcting for the spherical nature of resin particles, indicates the relative amount of BSA bound to the different resin samples throughout uptake (Fig. 6).

Figures 5 and 6 show a dramatic difference was found in the protein uptake rate of the different samples. Clean resin had the fastest uptake, followed by partially fouled, then extensively fouled resin. Partially fouled resin took approximately twice as long as the clean resin to reach the highest light intensity seen in the experiments, and fouled resin still had not reached this value after 85 min of loading. However, the amount of BSA bound to the partially and extensively fouled resin samples was approaching that of the clean resin at the end of the experiment. Partially fouled resin was at 98%, and extensively fouled resin at 83%, of the clean resin's capacity, and adsorption was still ongoing. This suggests that if the partially fouled or extensively fouled resin is challenged for long enough, it will eventually reach the capacity of the clean resin, or somewhere near this. For all three resin samples there was minimal difference between the shape of the intra-particle binding profiles. Some differences would have expected, either localized or in general, between the different resin samples had foulant been irreversibly binding to intra-particular binding sites, but this was not the case. In Figure 5, the peak close to the exterior boundary of the particle does become marginally wider and less defined with fouling, but this effect is minimal. This flattened region of the profiles in the fouled resin samples is approximately 3 µm in length, and may indicate that slightly less protein may be binding to this region as fouling worsens. These results are all in agreement with the hypothesis that the foulant forms a layer on the surface of the resin and does not significantly penetrate into the particles. It appears that as the foulant blocks access to the pore entrances, the available surface area where protein can diffuse freely into the particle therefore decreases, which introduces increased resistance to mass transfer. This causes the dramatic differences in uptake, but minimal differences in capacity which we saw between the resin samples. Intra-particle mass transfer thus does not appear to be a limiting step.

### Column Studies

Column breakthrough experiments were used for studying the effect of the foulant on resin performance. Scale down cycling studies provided fouled samples to measure BSA breakthrough over an extended range of flow rates. The use of BSA for breakthrough studies was not intended to replicate industrial process behavior. Instead, the BSA was used as an analytical tool to test the pore blockage hypothesis where resin fouling would hinder mass transfer into the resin, only allowing capacity of the resin to be recovered at high residence times.

**Figure 7:**
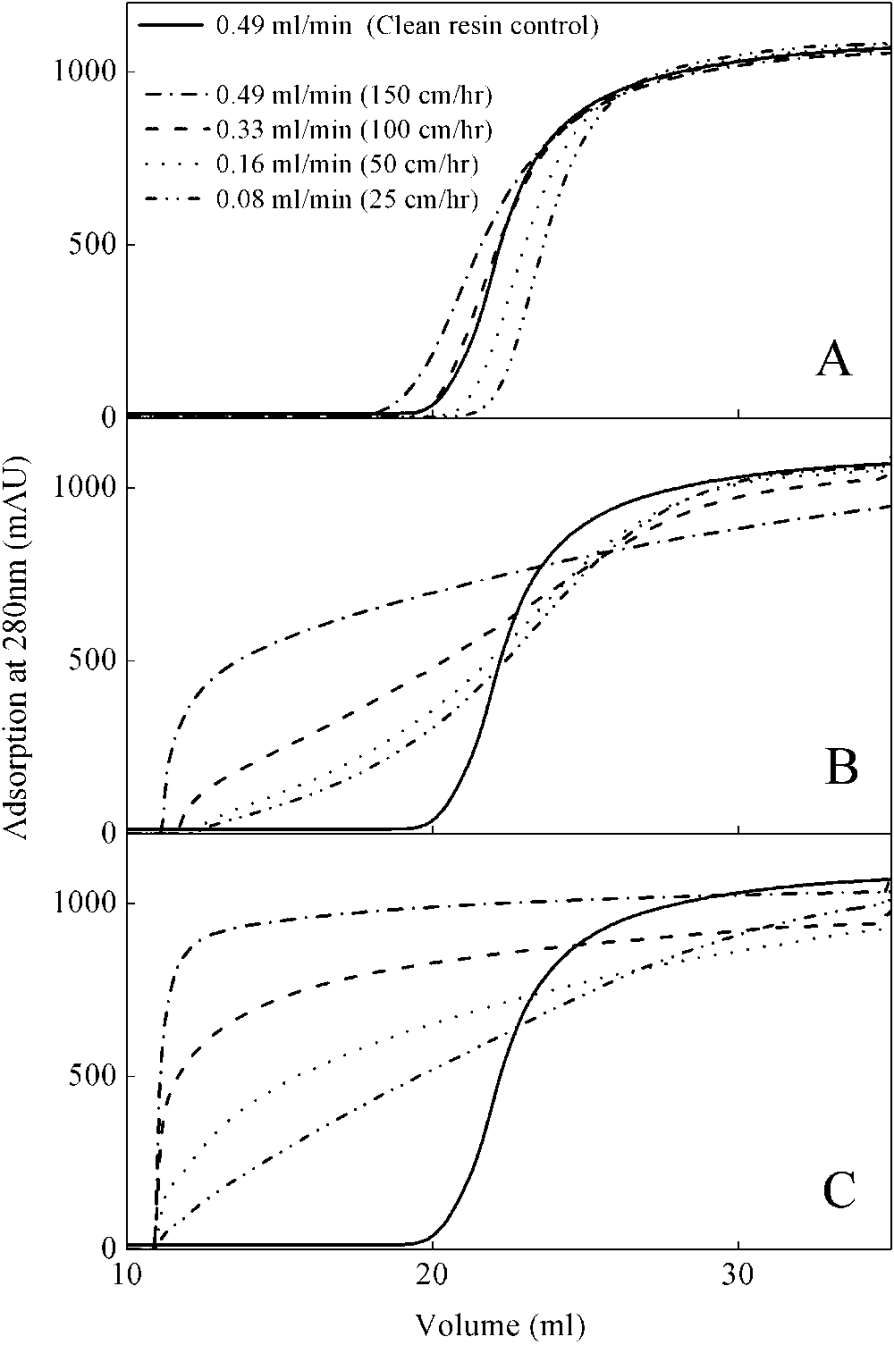
Breakthrough curves of 10 (mg/mL) BSA load in 0.05 M TRIS Base pH 9.0 at different flow rates on a 0.98 mL TMAE HiCap (M) column, 5 cm in length. Load phase begins at 10 mL. The column had been previously challenged with protein A peak containing 1 g (A), 3 g (B), and 5 g (C) of mAb. The control experiment using clean resin at 0.49 mL/min (∼150 cm/h) is shown for reference on each graph.

**Figure 8:**
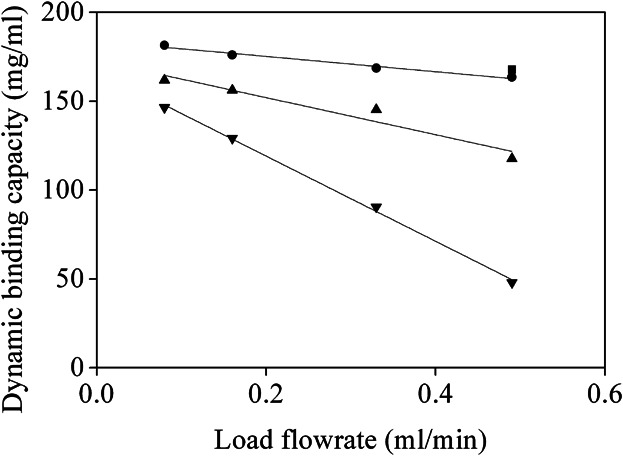
Fractogel® EMD TMAE HiCap (M) resin BSA dynamic binding capacity (at 90% breakthrough) as a function of load flow rate on a 0.98 mL TMAE HiCap (M) column, 5 cm in length. The column had been previously challenged with protein A peak containing 0 g ▪, 1 g •, 3 g ▴, 5 g ▾ of mAb. The load concentration of BSA was 10 mg/mL in 0.05 M TRIS Base pH 9.0. Data arrived from Figure 7.

Figure 7 shows that over the course of the column studies, both the shape and the position of BSA breakthrough changed drastically as cycle number increased. Figure 8 illustrates the corresponding decrease in dynamic binding capacity (DBC), which for the normal operating flowrate of 0.49 mL/min (∼150 cm/h), dropped by 71% over the course of the study. At this flowrate, Figure 7 shows that for clean resin, breakthrough begun after approximately 10 CV of material had been applied to the column. In contrast, when the resin was extensively fouled, onset of breakthrough was rapid, beginning after less than 1 CV, similar to what would be expected during operation in flow through mode with minimal protein binding.

The shape of the breakthrough went from sharp to diffuse as fouling progressed (Fig. 7), and indicated that the loss in capacity and rapid breakthrough observed at the end of the study was due to severe mass transfer resistance, rather than a decrease in capacity due to foulant binding in place of the protein molecule of interest. Breakthrough would have been expected to remain sharp if the mass transfer was not effected by fouling, which was not the case.

The data show that by decreasing the flow rate, the DBC lost due to fouling can be recovered (Fig. 8). Reducing the flow rate from 0.49 mL/min (∼150 cm/h) to 0.08 mL/min (∼25 cm/h) at the highest level of fouling (after 5 g of feed material had been challenged), resulted in the DBC increasing from 47 mg/mL to 146 mg/mL resin, a threefold increase. A linear equation with an *R*^2^ value of 0.997 was fit to the data from Figure 8. The *y* intercept of this equation showed that with the fouling levels experienced at the end of the study, the theoretical maximum DBC was 167 mg/mL resin, the same DBC as the control run conducted at the start of the study using clean resin. This further supports the hypothesis that the foulant is located on the particle surface, only penetrating the particle to a limited degree. The increase in time that particles are exposed to BSA at lower flow rates, enables the BSA to overcome the mass transfer limitations as a result of the fouling, thus restoring DBC to pre-fouled levels.

## Conclusion

Batch experiments, SEM and CLSM and small-scale column experiments are useful tools for characterizing fouling in chromatographic resin. In this study the foulant was shown to progressively build up on the particle surface using SEM. The batch and CLSM live uptake experiments were in agreement that the foulant reduced the uptake rate of the BSA reporter molecule. Little or no change in saturation capacity. The column study confirmed that binding capacity lost due to the foulant could be restored by decreasing the flow rate, providing further evidence to support the conclusion that the foulant is located on the particle surface, only penetrating the particle to a limited degree. The results suggest that progressive fouling of resin can severely impact the performance of chromatography columns. The knowledge gained from the use of these analytical methods increases process understanding, and thus provides for a better informed column development.
